# The Clare Hall Sanatorium Inquiry: Result

**Published:** 1918-11-16

**Authors:** 


### The Clare Hall Sanatorium Inquiry: Result.

THE DUTIES OF THE MEDICAL SUPERINTENDENT.

RECENTLY the Local Government Board held an inquiry into complaints by patients at the Clare Hall Sanatorium South Mimms, Middlesex.

This institution belongs to the Middlesex Districts Joint Smallpox Hospital Board. The inquiry was made by Dr. Coutts, one of the L.G.B. assistant medical officers, and the findings are summarised below :—

1. There was no conscious neglect of the patients, but, on the contrary, there was every desire to carry out their medical treatment efficiently, and to make the patients happy and comfortable. The Board does not find any foundation for the allegations (*a*) that there was actual neglect in medical attendance; (*b*) that no steps were taken by the Medical Superintendent to remedy complaints made by patients, and (*c*) that the general health of the patients suffered in consequence.

2. As regards the food of the patients, the Board finds that the endeavours made by the Sanatorium Authorities to provide variety in the food were not invariably successful. However, the Board is of opinion that all reasonable steps were taken by the Sanatorium Authorities to meet the difficulties.

3. As regards the cooking of the food, probably through carelessness, some of the food served was, on a few occasions, insufficiently cooked.

4. The Board is informed that the arrangement by which patients were required to delay eating their porridge at breakfast until all the patients were served was abandoned some time ago.

5. In general it would appear to the Board that the complaints made by the patients at the Sanatorium are to some extent the outcome of a spirit of restlessness and discontent, which may have been fostered by the failure of the Sanatorium Authorities, either to provide sufficient occupation, or to insist on the work provided being properly carried out.

6. As the Board takes the view that, although it is unnecessary to subject the patients to rigid disciplinary measures, it is nevertheless desirable to lay down definite rules for the conduct of the patients, and to make regulations as to hours of rest, recreation, and graduated labour. The reasons for the rules and regulations should be explained to the patients, especially as regards their relation to treatment, and steps should be taken to secure their due observance. For the latter purpose resort should, if necessary, be had by the Medical Superintendent to penalties varying from deprivation of minor privileges to immediate expulsion.

The Local Government Board makes the following suggestions with a view to removing any grounds which may exist for similar complaints in the future :—

1. To raise the standard of efficiency, it is necessary that the administration of the institution should be improved.

(*a*) Suitable rules should be framed for the conduct of patients at the Sanatorium and their strict observance insisted upon. In the event of a breach of the rules, the patient concerned should be warned, but if, in spite of such warning, he persists in refusing to abide by the rules, it would be in the interests of the other patients to dismiss him from the Sanatorium. (*b*) Suitable graduated labour for patients as part of their treatment should be provided and supervised. (*c*) More ground within the Sanatorium should be for the growing of vegetables. This would provide useful work for the patients and would secure greater variety in the diet. (*d*) The daily routine as regards rest, recreation, exercise, graduated labour, etc., should be definitely prescribed for all patients who are not confined to bed, and the routine so provided should be adhered to.

2. Under the direction of the Medical Superintendent, for the ordering and distribution of stores and the keeping of accounts in connection therewith, it would be the duty of the new officer to check the quantities received, and to ensure that stores of the requisite quality are obtained at the least cost.

3. In normal times contracts should be made for the purchase of the necessary supplies for use in the Sanatorium. This, apparently, has not been the practice at this Sanatorium.

4. The appointment of an officer to carry out the duties referred to in paragraph 2 above would enable the Medical Superintendent to devote more time to his clinical duties and to the more important administrative details of his office. The Medical Superintendent should guide the whole work of the Sanatorium, and in particular should deal promptly with causes of complaints as they arise.

